# Microbial terroir in winegrowing: revisiting the role of microbial biogeography in terroir expression

**DOI:** 10.1128/aem.01190-26

**Published:** 2026-07-20

**Authors:** Lena Flörl, Reid Griggs, Nicholas A. Bokulich

**Affiliations:** 1 Department of Health Sciences and Technology, ETH Zurich 311632, Zürich, Switzerland; 2 Stony Hill Vineyard, St. Helena, California, USA; Michigan State University, East Lansing, Michigan, USA

**Keywords:** fermentation, microbiomes, wine, microbial ecology, terroir, food

## Abstract

The concept of *microbial terroir* posits that microbial biogeography pre- and post-harvest contributes to regional variation in the sensory properties of wines and some other foods. Since the introduction of this concept, the field has rapidly advanced and accumulated strong evidence that regionally distinct microbial communities contribute to characteristic wine qualities. Biogeographic patterns of vineyard microbiomes by now have been documented across all major wine-producing regions, improving our understanding of microbial sources and dispersal, as well as the abiotic and biotic factors that shape community composition. Here, we summarize recent developments in *microbial terroir* research in winegrowing and address common misconceptions. Spatiotemporal environmental variation influences the composition of vineyard microbial communities, and we examine the specific reservoirs and contributing drivers of this diversity. We showcase how microbial biogeography, particularly the diversity of non-*Saccharomyces* species, influences fermentation dynamics and resulting wine characteristics and how this expression of *terroir* is further impacted by the winery environment and winemaking practices. Ultimately, we identify remaining knowledge gaps and discuss potential directions for future research to better understand the ecological and functional roles of microbial communities in viticulture and winemaking.

## WHAT IS *MICROBIAL TERROIR*?

*Terroir* describes the link between a product’s distinct characteristics and its geographic origin and has been a central principle in viticulture for centuries. Traditionally, *terroir* is understood to be influenced by both environmental and human factors, including soil characteristics, local climate, topography, viticultural practices, and regional choice of grapevine cultivar. This is reflected in regulations like protected denomination of origins (PDO), which are strictly defined over features such as distinct climatic conditions and geology, authorized cultivars, and often traditional management practices (1).

Growing recognition of microbial influence in viticulture and winemaking has given rise to the concept of *microbial terroir*, i.e., that microbial communities (microbiota) and microbial activities (collectively, the microbiome) pre- and post-harvest also contribute to terroir characteristics. This was facilitated, in part, by implementation of high-throughput DNA sequencing technologies to survey grape and wine microbiota, beginning in 2014 with the discovery that grapevine microbiota vary by region and cultivar across a range of viticultural zones in California, correlating with environmental characteristics (2). This was followed by further demonstrations that the chemical composition of fermented wines was associated with regional diversity of *Saccharomyces cerevisiae* strains (3), as well as with the overall composition of microbiota on the grapes prior to fermentation (4), suggesting that these microbial communities contribute to distinctive wine characteristics. Since then, an increasing number of studies have confirmed and expanded upon this idea, showing that regionally distinct communities can influence not only wine (3–5) but also other traditional fermented foods such as cheese or ham (6, 7). Microorganisms are increasingly recognized as an integral component of *terroir*, acting as an invisible and often still underappreciated contributor to product identity (8–10). Yet, some critiques and common misconceptions about *microbial terroir* have persisted (11), particularly regarding the spatio-temporal stability of winegrowing as a dynamic ecosystem, and recent work has started to fill in some of these knowledge gaps.

Significant progress has been made, driven by technological advancements that enable the integrative analysis of microbial communities, host genomics, and metabolite profiles. Only now are we beginning to unravel the complexity of vineyard microbial ecosystems and the mechanisms by which these communities influence wine characteristics. Here, we summarize the current state of research on *microbial terroir*, focusing on the environmental factors, host genotype effects, and viticultural practices that shape the microbiome and examine their potential effects on subsequent wine characteristics pre- and post-harvest. Ultimately, we identify remaining challenges and knowledge gaps and give an outlook over potential future research avenues.

## MICROBIAL BIOGEOGRAPHY IS A FUNCTION OF ECOLOGICAL AND EVOLUTIONARY PROCESSES

Studies investigating spatiotemporally diversity of *Saccharomyces cerevisiae* strains and their potential impact on subsequent wine fermentations date back to the 1990s (12–14). However, the collective influence of microbial biogeographic patterns was only recognized more recently (2, 3). Microbial biogeography describes and attempts to explain patterns of microbial diversity across space and time, as well as the ecological and evolutionary forces shaping them (15). The resulting patterns arise from a combination of processes that introduce diversity (dispersal, diversification) and local processes that modulate abundances (selection, drift) (see Box 1) (16).

Box 1. **A Mini-Glossary on the Mechanisms behind Community Assembly**

The forces behind community assembly can broadly be divided into stochastic (diversification, drift) and deterministic mechanisms (dispersal, selection).

**Stochastic Mechanisms – Diversification and Drift**. While **diversification** describes the “generation of new genetic variation,” **drift** is defined as the “stochastic changes in the relative abundances of different taxa within a community through time” (16). Together, these generate historical contingency, where the presence and abundance of taxa are shaped by evolutionary processes and random population fluctuations. In microbial ecology, the relative roles of stochastic and deterministic mechanisms are still debated and considered understudied (17, 18).

Deterministic **Mechanisms – Dispersal and Selection**. Studies of microbial biogeography typically focus on (relatively) deterministic mechanisms which include **dispersal**, describing the movement of microorganisms across space and time, and **selection**, encompassing the changes in a community based on differences in fitness under local conditions (16, 17). Both of these mechanisms are examined further in the following sections.

A **Note on Dispersal vs Transmission**. The term dispersal is typically used in the context of large-scale biogeographic patterns, while **transmission** describes the direct transfer and establishment of microbes between hosts or from the environment to a host (19). As most microbiome studies use cross-sectional study design and do not sample longitudinally or repeatedly, we often cannot differentiate between true residents and transient microorganisms. This is a known issue in phyllosphere microbiome research, as, for example, ‘contamination’ from airborne mushroom spores may be misinterpreted as resident fungi (20).

Dispersal varies considerably between kingdoms and among taxa based on differences in morphology, dispersal mechanisms, and lifestyles, which collectively determine if and how far microorganisms are dispersed (21). For example, fermentative yeasts need to be transported to new sugar sources and rely on vectors like insects or wind for dispersal (22). Morphological traits like spore formation enhance survival during dispersal, while interestingly, microbial size or mass do not necessarily predict dispersal distance (21, 23). Hence, dispersal limitations play a critical role in structuring abundance, richness, and evenness in microbial communities (24). Dispersal limitation effects appear to shape microbial biodiversity patterns at both local and regional scales (25–28), explaining microbial community heterogeneity both within individual vineyard plots (29) as well as between vineyards (30).

Biogeographic patterns also reflect ecological processes such as drift and selection (see Box 1) that drive community structure and the evolution of taxa. An example of environmental selection is local substrate availability, pH, or oxygen availability, which can alter competition dynamics, leading to shifts in community structure. In vineyards, site-specific factors influencing berry ripening and sugar availability directly influence microbial community composition (31). Successful colonization also depends on the ability of microbes to survive and compete in a new environment, which is constrained by the metabolic plasticity and genomic repertoire of taxa. Notably, microevolutionary changes (diversification, see Box 1) can lead to regionally distinct species or strains, and while communities may appear similar at high taxonomic levels (e.g., phyla), they can differ significantly at higher resolutions due to specific niche adaptations (32). This highlights the importance of considering the actual resolution of analysis when comparing closely related habitats. Therefore, the observed distance decay relationships, where locations which are physically closer harbor more similar microbial communities, can be understood as a proxy for niche separation along environmental gradients. This is important to not convolute implications of the traditional concept of *terroir* with *microbial terroir*, as microbial communities are obviously not strictly delimited by human-defined geographical boundaries.

Vineyards offer diverse microbial habitats which are shaped by complex interactions between abiotic and biotic factors, leading to distinct microbial community patterns both within and between geographic locations. Therefore, the scale of observation matters as, for example, intra-vineyard soil bacterial communities can vary more than even between vineyards (33, 34), while for grapes, distance decay relationships are more pronounced when comparing across larger spatial scales (26, 27, 30, 35). Soil and permanent plant organs like bark exhibit relatively stable microbiota across years, while short-lived ephemeral organs such as leaves, flowers, and berries show high spatial and temporal variability, shaped largely by dispersal (2, 4, 30, 36, 37).

To summarize, microbial biogeography is strongly shaped by the processes of dispersal and selection. However, dispersal alone does not alter biogeographic patterns unless microbes successfully establish themselves (Dispersal vs Transmission, see Box 1) (15). The effectiveness of this transmission, in turn, is additionally influenced by various abiotic and biotic factors, which we will discuss in the following sections.

## RESERVOIRS AND TRANSMISSION OF MICROBES IN VINEYARDS

In the context of *microbial terroir*, it is relevant to understand where microorganisms in vineyards come from and how they get there. Griggs et al. (22) provide a comprehensive review on the topic, and key mechanisms are summarized here. Fundamentally, microbial transmission can occur via two main routes: vertical transmission, where microbes are inherited through propagation material such as seeds or grafts, and horizontal transmission where microbes are acquired from the surrounding environment. In viticulture, vertical transmission is mostly relevant in the context of viral pathogens (38), and the vast majority of vine-associated microbes originate from environmental sources and are dispersed via wind, insects, animals, or human activity (22, 39).

**Fig 1:**
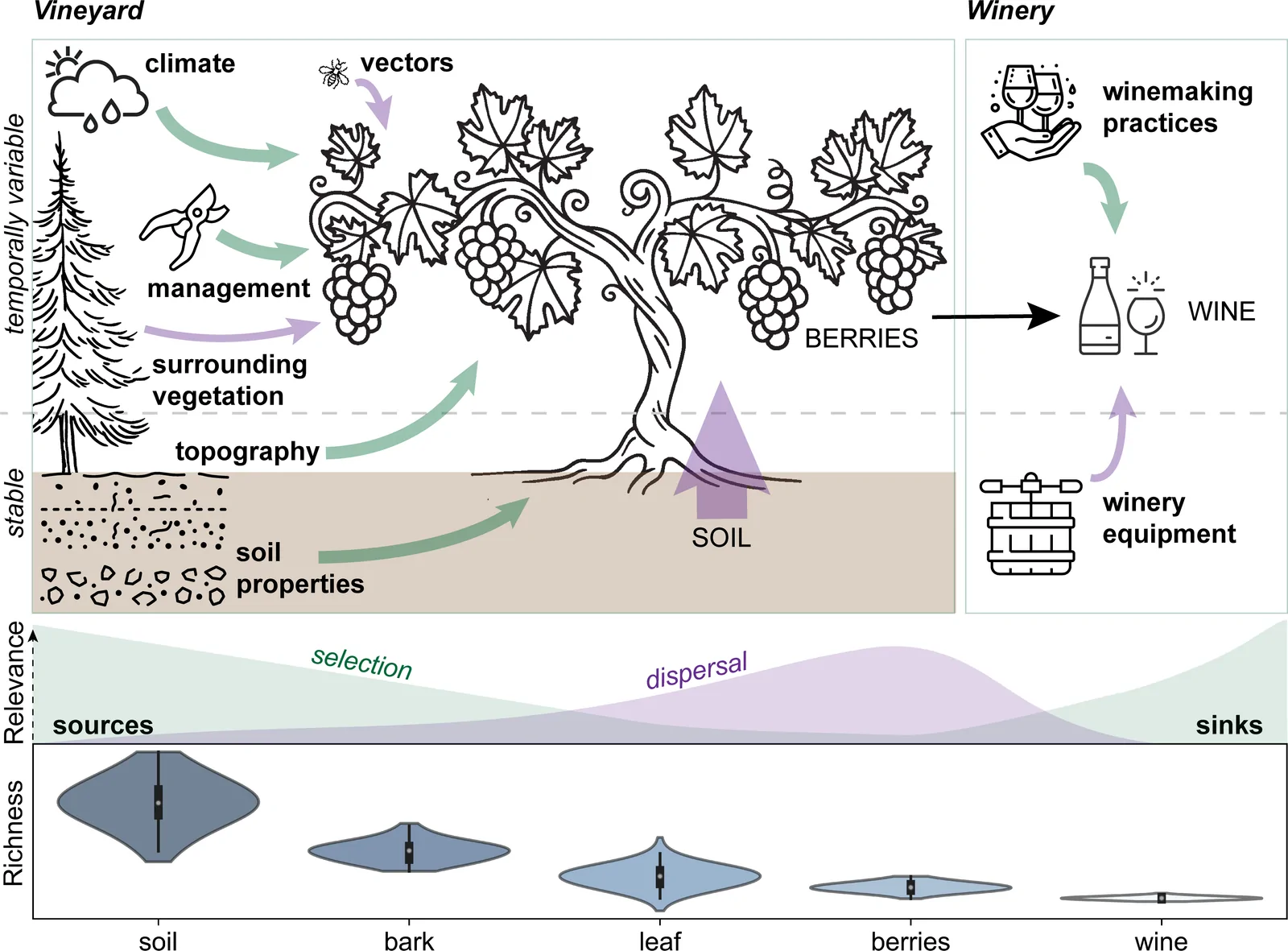
Abiotic drivers of microbial diversity and key microbial reservoirs in vineyards. Soil is a major reservoir of high microbial diversity (violin plots showcasing differences in richness at the bottom), primarily shaped by persistent environmental selection (green arrows) such as differences in soil properties and topography. Soil and bark are the main sources (purple arrows) for microbial colonization of annual, ephemeral plant organs like leaves and berries, which are primarily shaped by dispersal and secondarily through environmental selection such as variable climates (Relevance: estimated relative relevance of ecological forces on community assembly, approximated also by the thickness of the arrows) (36, 37, 41). Berry-associated communities are then transferred to the winery, where microbial composition is further shaped by selection through distinct winemaking practices and some dispersal of resident microbes from equipment, which ultimately collectively impacts wine characteristics. While vineyards are open systems with continuous microbial dispersal, wine fermentation is a comparably closed system in which fermentation conditions and the dominance of *Saccharomyces cerevisiae* impose strong selective pressures.

Among environmental sources, soil plays a central role as a microbial reservoir in agricultural settings. In viticulture, it is widely recognized as a key determinant of vine health and grape quality, and in the context of *microbial terroir,* it is considered a primary reservoir for microbial diversity, especially for the annual re-colonization of ephemeral plant organs like leaves, flowers, and berries (22) (Fig. 1). Endophytes, which live within plant tissues, inhabit many plant organs but especially the root and vascular systems and mostly enter the plant from soil (40). On the contrary, epiphytic microbes reside on plant surfaces and are derived from various sources, including soil, surrounding vegetation (e.g., forests or cover crops), or transmission among plant compartments (e.g., bark to berries, leaves to berries), as well as insect vectors or humans (22). These epiphytes include the microbes that will have a direct impact on the resulting wine since fermentative yeasts and bacteria that colonize the fruit surface can seed the fermentation as fruit travels to the winery.

Of particular interest is the transmission and strain-level diversity of fermentative organisms, such as *Saccharomyces cerevisiae,* as well as other yeasts and lactic acid bacteria, which directly contribute to fermentation dynamics and wine sensory profiles (32, 42, 43) and exhibit distinct phenotypic traits including fermentation performance and aroma production. *S. cerevisiae* and *Hanseniaspora uvarum*, the dominant non-*Saccharomyces* yeast on grape berries, exhibit substantial variation in abundance of strains (43, 44) and genetic variants across vintages, vineyards, and wineries (45). Prevalence of both species is also differentially associated with cultivar and grape berry chemistry and influenced by vintage and microclimatic conditions (30, 44, 46, 47). In the case of *S. cerevisiae*, strain distribution was further shown to be limited by their dispersal, rather than in what specific niche within a vineyard or surrounding area a strain has been isolated from (48).

As outlined above, soil is a major reservoir for yeast and induces sporulation in *S. cerevisiae*, indicating a bi-directional transmission between soil and berries through seasonal cycles (49). Mutualistic interactions with insects also play a key role in yeast dispersal and interannual persistence. Many yeasts produce specific volatiles that attract insects, facilitating their transport between fruits and enabling overwintering, as yeasts can be vertically transmitted to offspring (50). Furthermore, the transmission and prevalence of fermentative yeasts have been strongly linked to human activity (3). Additionally, different vineyard management practices appear to influence the prevalence of fermentative species (51, 52).

To better understand the complex interplay of these factors, the following sections will explore in more detail the environmental, biological, and anthropogenic drivers of microbiome assembly in grapevines.

## SITE-SPECIFIC ENVIRONMENTAL FACTORS ARE THE PRIMARY DRIVER OF DIFFERENCES IN VINEYARD MICROBIOMES

Microorganisms are dispersed and the environment selects for which strains establish themselves and persist, thereby shaping both the composition and diversity of microbial communities, as well as their ecological functions and interactions. As such, the site, as a confluence of various environmental factors, is the overarching factor structuring microbial communities in vineyards (2, 22, 27, 30, 53). However, disentangling the influence and interactions of individual environmental factors remains notoriously difficult to control for in vineyard settings. While much research has been conducted in model plants like *Arabidopsis* (54, 55), perennials like grapevines are less studied due to their long lifespans and lengthy establishment periods. However, grapevines are an interesting model as they are a long-lived perennial and highly sensitive to environmental variation. The same cultivar in different locations, even within the same region or a single vineyard, can yield distinct metabolite and gene expression profiles (phenotypic plasticity) (56–58), which is further expressed in different wine sensory characteristics (59). These differences coincide with variable microbial communities (4, 30) and suggest that microbial colonization of grapevines may be a factor in shaping the plasticity of grape and wine metabolomes, as a complex phenotype consisting of both plant- and microbial-derived metabolites.

The site specificity and temporal stability of vineyard microbiota depend on the specific habitat. Some habitats, such as soil and bark, are shaped by persistent environmental influences and host more temporally stable microbial communities (60). Temporal dynamics, such as ripening stages or vintage effects, further contribute to this site specificity (30). Accordingly, soil communities are commonly found to be strongly associated with pH as well as topographical features like altitude and slope, which often correlate with other soil properties (30, 33, 34, 61). Particularly, soil chemistry appears to be a stronger predictor of microbial community composition than geographic distance alone (62), suggesting that microbial selection predominates over dispersal (36). As mentioned before, this can lead to higher variability of grapevine microbiota within than between vineyards due to small-scale heterogeneity in soil chemistry, topography, water retention, pH, and temperature (33, 34). Yet, it is important to note that these variables are not static either. For example, pH—which is commonly considered a key variable in soil systems—fluctuates seasonally and is influenced by variable factors such as plant growth (63).

In contrast, microbial communities on ephemeral, above-ground plant compartments, such as leaves, flowers, and berries, are more susceptible to variable environmental conditions as they are more exposed and, hence, can exhibit greater temporal variation. These annually newly formed organs are colonized by microorganisms from the surrounding environment, and priority effects from early colonizers and transient microbes could substantially shape the trajectory of community assembly (64, 65). Insect vectors also play an important role in transmitting many microorganisms, including fermentative yeasts (50, 66), and hence the timing and spatial density of insect visits at different stages of grape growth may add further complexity to microbiota succession dynamics. As such, stochastic dispersal effects likely contribute to differences in community composition between years and potentially to small-scale spatial heterogeneity within vineyards.

Local and inter-annual climatic differences play a major role in shaping these communities, as shown by the influence of even subtle microclimatic effects as well as strong vintage effects (30, 46, 67) and high spatial heterogeneity, even within individual vineyard plots (29). Notably, the extent of vintage effects appears to vary by region, as some studies only report minimal inter-annual community differences (68). Furthermore, the scale of the analysis and the microbial taxa examined matter, as, for example, bacterial communities seem to be more variable between years than fungi (2). A notable recent insight is the concept of temporal decay, describing how microbial communities of berries appear more similar between consecutive years than general year-to-year variation (30). This is important to highlight, as it underscores a common misconception: microbial communities and their influence on *terroir* expression are not fixed, as they respond to annual weather variability. This raises interesting questions about the rate and extent of community fluctuations, particularly in the context of long-term climate trends. This will require further studies, including studies of the temporal variations in wineries, which were also shown to fluctuate both seasonally and annually (69, 70) in response to indoor environmental conditions (71) and are key microbial reservoirs for wine fermentations (45).

The presence of inter-annual variation in microbial communities is occasionally cited as an argument against the theory of microbial terroir (11). However, it is important to consider that temporal variation is also a key, recognized characteristic of wine quality, encompassed in the concept of vintage. In terms of both wine quality and microbiota composition, temporal variation is not random but reflects responsiveness to annual variation in environmental conditions (2, 28, 30) as well as pre- and post-harvest practices. Hence, these properties mirror each other and represent gentle, annual fluctuations that may be interlinked (as the theory of *microbial terroir* would posit). As the environmental influences shaping microbial communities are complex and difficult to disentangle, more rigorous and large-scale studies integrating multiple layers of data are therefore essential to uncover the contributions of individual factors (30).

## THE HOST GENOTYPE EXERTS A DISTINCT, CONTEXT-DEPENDENT INFLUENCE ON ASSOCIATED MICROBES

A key biotic factor influencing microbiota selection is the grapevine itself, as it actively recruits and modulates its microbial symbionts (47). Different plant genotypes harbor variable microbial communities, which are structured along the continuum of the host phylogeny (72, 73). In grapevines, different cultivars have been reported to host distinct microbial communities in berries (2, 74–78), the rhizosphere (79), and leaves (76). The practice of grafting cultivars on variable rootstocks adds another layer of complexity, as this makes a single vine containing two genotypes. This combination influences the gene expression and phenotypes in both the scion and rootstock and was shown to vary with environmental conditions (80). These interactions affect recruitment of rhizosphere communities (81–84), as well as endophytes (85), and may also influence communities in leaves and berries, including key fermentative species such as *S. cerevisiae* (51, 82).

Importantly, the influence of the host genotype on microbiome assembly is mediated through phenotypic traits, such as variable morphology, physiology, metabolism, and stress or immune response (39). Grapevines can exhibit a high degree of phenotypic plasticity, which is the capacity of a single genotype to express distinct phenotypes under different environmental conditions, and consequently, the effect of the genotype on the microbiome is not static either but similarly depends on both the time and environmental conditions (39, 86, 87). As microbial communities themselves are strongly shaped by these abiotic factors, the influence of genotype is highly confounded, leading to distinct genotype-by-environment interactions (2, 30, 88). On a fundamental level, this relationship between genotype, environment, and associated microbes has long been recognized and is reflected in traditional knowledge and cultural practices, as well as in the context of disease susceptibility. For example, cultivars exhibiting a tight cluster morphology are known to be more susceptible to cluster or sour rot diseases in humid climates due to the retained moisture (89).

**Fig 2:**
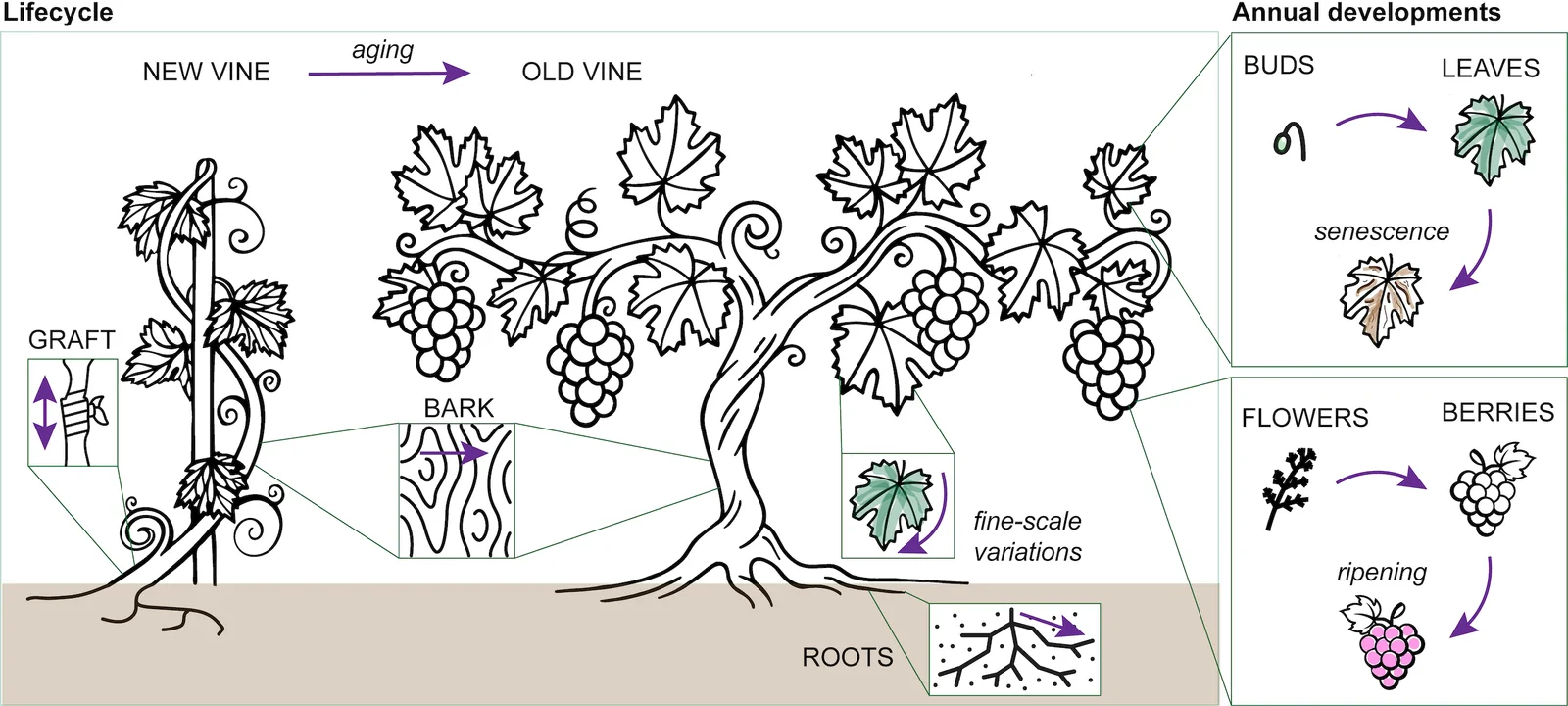
The biotic influence grapevines exert on associated microbial communities changes over their lifecycle as the vine ages, as well as in the annual developments (time-dependent effects of the genotype on microbiota are indicated with purple arrows). As long-living perennials, grapevines are valuable models to study the variable influence of the host genotype over time (e.g., in initial grafting, or with bark or root aging). Notably, the genotype influence can also vary between and potentially within different plant compartments, as well as over the season. This is particularly relevant for annually newly developed organs. These developmental changes, e.g., the formation of leaves, flowers, and berries, lead to the assembly of distinct communities (98). Interesting examples include berry ripening, which leads to drastic shifts in microbial diversity, and leaf senescence where the plant tissue becomes attractive to saprophytes which ultimately disperse back to the environment.

Just as variable genotypes create temporally distinct morphological features, the microbial communities in these respective niches also exhibit variable responses to the host age or annual variations (39, 90). Understanding the temporal dynamics of genotype effects on the microbiota, therefore, requires consideration of the vine’s life stage, from initial grafting to planting and aging, as well as the annual developments of its organs (91) (Fig. 2). Such age-related differences are reflected, for example, in an increased fungal diversity in bark as the vine ages, likely due to changes in bark surface and increased water retention (92, 93). Additionally, annual developmental timing (phenological stages) causes systemic physiological changes (39). For example, as leaves age, their wax composition, cuticle structure, available nutrients, and immune responses change, which directly influence microbial communities, and result in seasonal as well as intra-canopy variations (91). Particularly relevant in viticulture is the differential effect of the host on berry communities, which a plethora of studies report to drastically shift with ripening (30, 47, 94–97). As berries ripen acidity and pH decrease, while sugars accumulate, which causes the microbiome to transition from initial leaf-like communities to one with lower diversity but more fermentative organisms at peak ripeness (22). Yet, as recently shown, different genotypes still exert a distinct influence on microbial community assembly across this process (47). It is also noteworthy that these plant compartments can exhibit fine scale phenotypic variation. For example, within a leaf there may be a morphological or physiological gradient from base to tip and root systems are highly heterogeneous, forming distinct sub-niches (39) (Fig. 2).

However, while associations between host genotype and microbiome recruitment have been reported in other systems (99–101), the underlying genetic and physiological mechanisms in grapevines remain less explored. A first study performing genomic mapping of berry microbial communities in first generation (F1) crosses revealed robust associations between specific hotspot *loci* with multiple taxa, among them key fermentative yeasts and potential pathogens (47). Conducted across two vintages and different ripening stages, this gives first insights into variations as well as conserved genetic mechanisms influencing microbiome assembly under differing environmental and developmental conditions. Future studies could focus on analyzing these retrieved *loci* and associated genes, as well as the identified key “hub” microbes, which appear central to microbial co-occurrence networks and closely linked to the host genotype. Notably, even among this full-sib progeny, the different genotypes recruited distinct microbial communities, highlighting the expectedly far greater differences between cultivars of more divergent genomic backgrounds.

## HUMAN PRACTICES SHAPE MICROBIAL DIVERSITY IN VINEYARDS

The third major category of factors influencing microbial diversity in vineyards is vineyard management practices, which are similarly confounded by environmental conditions. Practices like tillage, cover cropping, and fertilizer or pesticide input can modify soil properties (e.g., nutrient availability) and thereby shape soil microbial communities (67, 102). However, the extent of these effects is modulated by soil type, which further complicates efforts to disentangle management from environmental influences (103). While soil microbial communities are often found to be directly affected by different management practices (26, 104, 105), the translation to the fruit microbiome is less clear (67, 106). This is likely because the microbial communities of ephemeral organs are more shaped by variable environmental factors and exposed to dispersion. Fungicides are routinely used to control phytopathogenic fungi and can alter microbial communities on grapes and leaves, yet their specific effects depend on the product and timing (105, 107–109). Another way in which management practices may influence microbiota is through their impact on plant stress (e.g., irrigation and drought stress) (110), which contributes to differential recruitment of microorganisms (47, 111). Apart from active viticultural management practices, any anthropogenic activity such as traffic on adjacent dirt roads creating more dust can locally increase microbial diversity (29). The sensitivity of the berry microbiome to minor environmental variations is further reflected in the influence of vineyard design. For example, row orientation can modulate microclimatic conditions, including temperature, light exposure, and humidity, and was shown to exert a distinct influence on berry microbiome assembly (88). While the influence of different management practices is, therefore, difficult to disentangle, they do exhibit a distinct, secondary, and highly location-dependent effect on microbial communities, as was recently shown for soil and even berries (52).

## MICROBIAL BIOGEOGRAPHY INFLUENCES WINE CHARACTERISTICS

**Fig 3:**
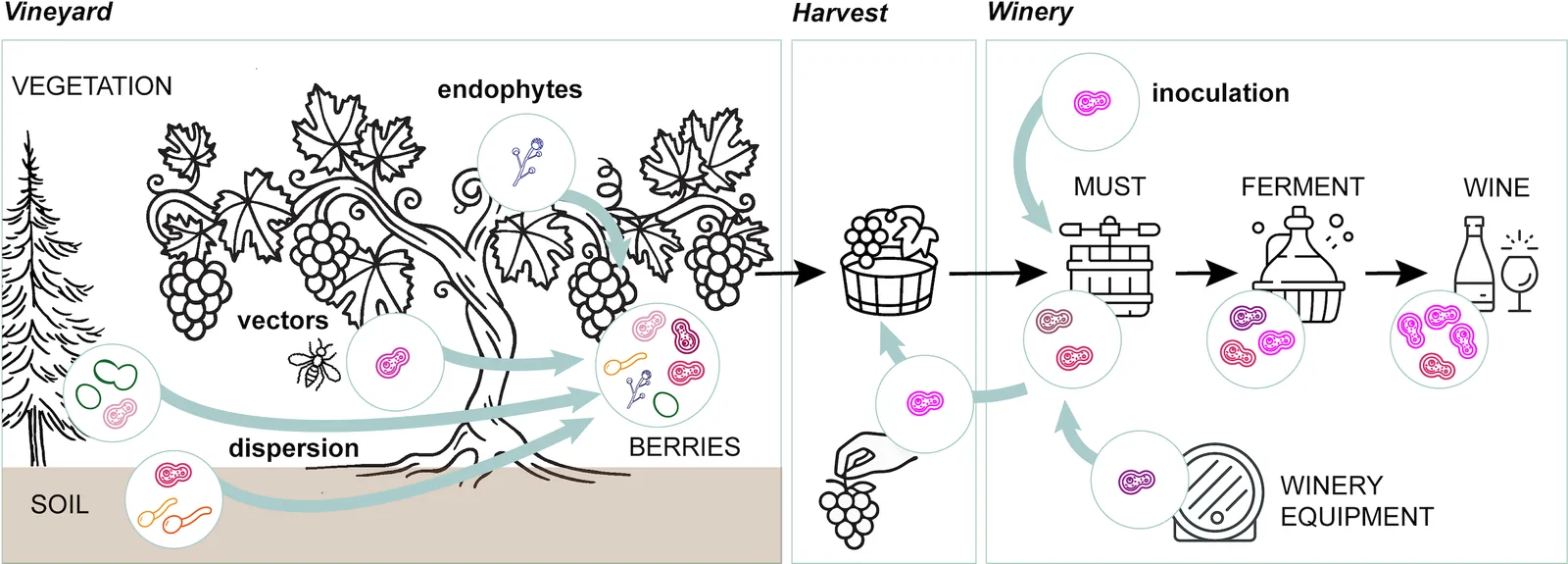
The sources of and selection for fungal taxa pre- and post-harvest in wine making. Berry-associated fungal communities are introduced to vineyards through dispersal by wind from surrounding vegetation or the soil, direct transmission through vectors (e.g., fruit flies, wasps), or potentially transmitted through the xylem as endophytes (5, 22). Different ecological niches, such as the soil or berries, select for different species and strains. Taxa can also be introduced through human activity, e.g., *Saccharomyces cerevisiae* strains can be transmitted back from wineries through practices like applying lees or pomace to vineyards. In the winery, the berry-associated species are a main source of microbial diversity in the grape must in addition to transmission of resident fungi from the winery equipment as well as direct inoculation with yeast. During the fermentation process, the diversity decreases, and eventually, the community is dominated by single strain(s) of *S. cerevisiae* (112).

Microbial communities in vineyards vary across space and time and are influenced by various environmental conditions, host genotype, and human practices, but what functional consequences do these communities contribute to the resulting wine characteristics? Microbial communities in vineyard systems can fundamentally exert influence both pre-harvest, by modulating vine physiology, health, and grape chemistry, and post-harvest, by directly contributing to fermentation (see Fig. 3).

Regarding the pre-harvest influence, soil microbial communities play a central role in nutrient cycling and in shaping plant health (113). In the grapevine, variable soil microbiomes and specifically plant-growth-promoting microbes influence resulting berry and wine qualities (22). Beyond that, recent studies also show that berry-associated microbial communities affect grapevine gene expression and volatile compound production, suggesting a link to wine aroma (114).

Plant pathogens are another intensively studied, major microbial influence on grapevine phenotype, yet the extent to which they contribute to wine characteristics is still unclear. Multi-species complex diseases such as sour rot or infections of single pathogens like *Botrytis cinerea* can spoil entire harvests or alter the aroma profile of wine (115, 116). *B. cinerea* is an interesting example, because if it infects ripe berries under specific climatic conditions (moist nights and dry days), it is limited to growing on the berry surface, causing noble rot, and the resulting desiccated berries are used to produce high-quality dessert wines (117–119). Such an infection substantially alters the grapevine’s physiology, including gene expression and metabolism of phenolics, lipids, amino acids, and volatiles, and directly affects the resulting grape quality (120–122). Further downstream, a *B. cinerea* infection can be detrimental, as for example, the fungus secretes, among other enzymes, laccases which oxidize grape polyphenols and can lead to undesired browning and sensory defects during winemaking (123, 124).

In reality, many putative pathogens can be detected even in healthy grapes and have been shown to co-occur with fermentative yeasts (47, 94, 125), likely due to the increased sugar availability of damaged grapes which has been shown to increase abundance and diversity of yeasts (43). These co-associations between pathogens and yeasts have also been reflected by associations with the same regions of the vine genome (47). Apart from fermentation beneficial yeasts, some spoilage organisms, e.g., *Gluconobacter*, are commonly detected on berries which can proliferate during fermentation and cause undesired flavors, while at low concentrations can increase perceived complexity of wine (126, 127).

Fungi play a central role in the expression of *terroir* of wine (26, 42, 46) partly because alcoholic fermentation is primarily driven by yeast and because fungi exhibit stronger evidence of dispersal limitation than bacteria (25, 47). This was corroborated by studies of spontaneously fermented wines, which showed that variable fungal communities substantially contribute to wine aroma and chemistry, while bacterial diversity had comparatively minor effects (5, 128). This was recently also demonstrated in inoculated wine produced without the addition of SO₂, thereby allowing for bacterial growth, where various fungal taxa were still more strongly associated with vintage distinct metabolites in resulting wines (30). Notably, although most fungal species become inactive during early fermentation (129), their presence may still impact wine chemistry through extracellular enzyme activity or cell lysis. For instance, commercial enzymes used in winemaking to improve color and aroma are often derived from *Aspergillus* species, which are also common grape commensals (130). Yet disentangling the roles of microbial metabolism, interspecies competition (e.g., killer toxin production), and cell lysis remains a key challenge in understanding how microbes influence wine aroma (96, 131). Furthermore, the potential roles of non-fermentative microbes, such as endophytes or filamentous fungi, remain poorly understood; their abundances are highly variable with environmental conditions (30) and host genotype (47), so an enticing future direction will be to assess what role these may have in grape health, fermentation dynamics, and wine quality.

## THE VARIETY AND ROLE OF NON-*SACCHAROMYCES* SPECIES IN WINEMAKING

All wine fermentations are eventually dominated by *S. cerevisiae*, yet even in stable, industrial fermentations, the initial community variation was shown to affect the fermentation trajectory and sensory outcome (132, 133). Trace-level metabolites (ppm–ppt range) produced or released by low-abundance microbes can significantly alter wine aroma (96). The presence of non-*Saccharomyces* yeasts in the initial must is particularly associated with increased aromatic complexity and *terroir* expression in spontaneously fermented wines, and a recent review of the literature catalogued 293 different non-*Saccharomyces* yeast species found in vineyards or wineries globally (43). Notably, while some of these species exert characteristic effects in wine fermentations (134), strain variation within fermentative organisms substantially contributes to distinct wine characteristics, as has been shown for *S. cerevisiae*, *Hanseniaspora uvarum*, *Oenococcus oeni,* etc. (3, 44, 135–137). This strain diversity arises among others, from different winemaking activities which have been shown to shape the evolution of fermentative species (138), among them *S. cerevisiae*, *H. uvarum*, and *Torulaspora delbrueckii* (136, 139, 140). Importantly, most microbial terroir studies to date have used short-read amplicon sequencing targeting short segments of the *rrn* operon (e.g., of 16S rRNA gene region or internal transcribed spacer amplicons). This approach lacks sufficient resolution to differentiate strains, and hence, much of this ecologically and functionally important diversity remains hidden and starkly constrains our interpretation (32).

Hence, many key open questions remain, including the origin and establishment of microorganisms, as well as the relative contribution of vineyard vs winery-associated microbiota. In addition, the role of microbial taxa not considered fermentative, and the impact of strain-level phenotypic variation within non-*Saccharomyces* yeasts and bacteria might explain markedly different community function and fermentation outcomes and deserve deeper consideration in future research.

## THE INFLUENCE OF WINEMAKING PRACTICES AND THE WINERY ON TERROIR EXPRESSION

Different winemaking practices can further contribute to (or reduce) the expression of *microbial terroir*, as they directly influence microbial dynamics in the fermentation and beyond. These include direct control through the inoculation with commercial yeast strains or manipulation of the fermentation niche through the addition of sulfites (SO_2_) to hamper bacterial growth (132, 141, 142), temperature control of the fermentation vessel, sanitary state of the receiving winery, and pre-fermentation practices like gas blanketing (69). Cold soaking, originally used to increase color and aroma extraction from grape skins, is also known to promote growth of non-*Saccharomyces* yeasts including *Hanseniaspora* spp. and potentially supports the expression of regionally distinct aromas (137). Notably, many of these conventional winemaking interventions aim to suppress the activity of native microbial communities and thereby limit *microbial terroir* expression, whereas enhancing such expression is a key motivation of winemakers relying on spontaneous fermentations. The specific impact of these applications on microbial diversity and function is highly context dependent; for example, the addition of sulfite influences different microbial clades depending on the dosage, pH, the presence of other compounds binding sulfites, as well as the inoculation strategy (143, 144). Additionally, practices such as cold soaking, settling, and micro-oxidation have been shown to affect microbial activity (137, 145, 146). While individual studies show how certain interventions can enhance or suppress specific taxa and thereby influence aroma formation and expression of *terroir*, in reality, winemaking remains a highly complex and variable process. Winemakers are drawing from individual experiences and preferences and make decisions that are highly context-dependent, shaped by physical constraints, culture, and tradition. Some of the cultural practices that are common may impact wine, in part, through microbial mechanisms, whether known to their practitioners or not. Moreover, wine production involves managing a constantly evolving niche from crush to the bottle and is invariably a multispecies process; much of the winemaker’s role is to control the rate and type of microbial activities during fermentation and maturation to calibrate the appropriate level of microbial expression.

While grape-associated microbial communities are the main source of fermentative diversity, particularly in spontaneous fermentations, microbes can also persist on winery surfaces as reservoirs for microbial transfer between fermentations (69). Although further research is needed to clarify the specific taxonomic and functional contributions of microbes originating from the vineyards vs those from the winery, it has been shown that winery resident microorganisms do not override the site-specific imprint of the respective vineyard microbiome (45). The winery can act as a reservoir for the transmission of microorganisms across fermentation batches and years (45, 69). Wineries harbor diverse bacterial and fungal communities on equipment surfaces (45, 69), including interannually persistent strains of *S. cerevisiae* which were shown to be related to their relative vineyard sources (147, 148), as well as significant populations of molds in the air (149, 150). These microbial communities on winery surfaces thereby fluctuate throughout the year. At harvest, the sudden availability of sugars promotes proliferation of resident yeast (69), while the introduced new microbes cause the equipment surface communities to largely reflect those of incoming fruit (45). Factors like the ventilation and cleaning practices are further expected to influence these winery- associated microbial communities (69, 149), and indoor environment plays a key role, with factors like temperature and humidity selecting for species growth on equipment and non-processing surfaces (71). We, therefore, propose expanding the understanding of *microbial terroir* to include wineries and to further study the interactions with the respective environmental settings and human practices.

## OUTLOOK

**Fig 4:**
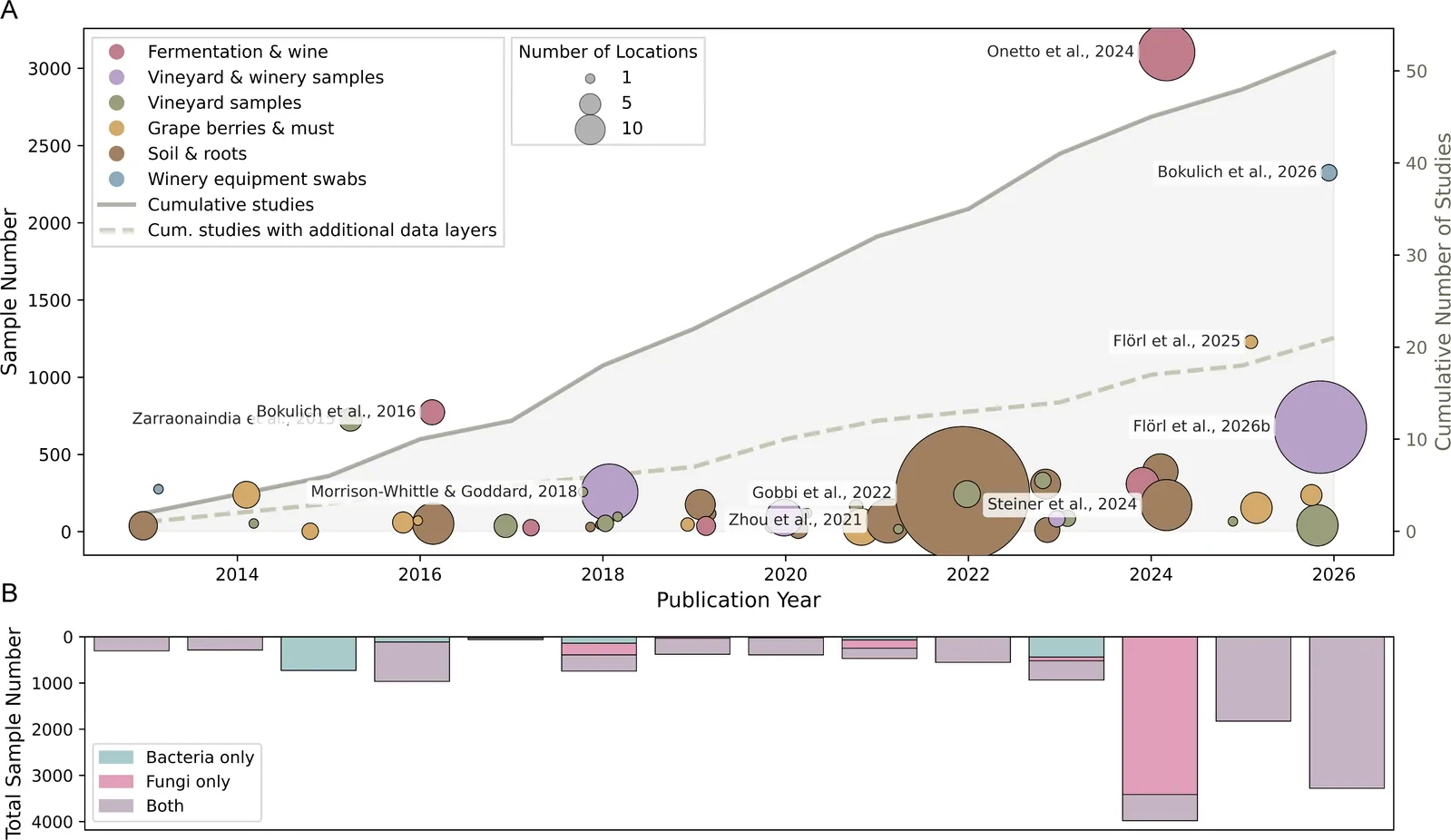
Summary of 54 studies referenced in this review, which profiled microbial communities of vineyards and wineries using metabarcoding (see full table at 10.5281/zenodo.20282193). (**A**) Bubble plot of publication year vs sample number per study, with the bubble size indicating the number of sampled locations, and color denoting the sample type (fermentation & wine, both vineyard & winery samples, various vineyard samples including leaves, bark, etc., grape berries & must, soil & roots, and winery equipment swabs). Studies including more than 20 locations or more than 500 samples are labeled. The solid line shows the cumulative number of selected studies over the years, and the dotted line shows the cumulative number of studies that include additional data layers such as metabolomics or shotgun sequencing. (**B**) Cumulative total sample numbers by year, with stacked bars showing whether studies analyzed bacterial communities, fungal communities, or both.

For centuries, viticulture and winemaking practices have (sub)consciously shaped microbial communities, but only now are we beginning to unravel these complex interactions and their contributions on resulting wine characteristics. Similarly, an ever-increasing number of vineyard and winery studies, which are also growing in scale (see Fig. 4 and the supplementary table at 10.5281/zenodo.20282193), are resolving individual components of microbial terroir, yet exceptionally few integrate these dimensions to deliver comprehensive system level insights. Hence, there remain immense gaps in our understanding of the causal mechanisms underlying the influence of environment, vineyard management, and the grapevine on microbial activity pre- and post-harvest. As microbial communities are highly dynamic and their behavior strongly context-dependent, future research will need to further disentangle the respective influences of spatial and temporal factors in microbiome assembly and function.

Advancing this research will require improved sampling designs, particularly spatially aware (29, 151) and longitudinal approaches, as well as the careful consideration of confounding factors (e.g., cultivar identity, grapevine age), which are still largely overlooked in vineyard microbiome studies. While bioinformatic advances, such as the shift from OTU- to ASV- and k-mer-based analysis (see Fig. 4), already improved taxonomic resolution, the increased availability and emergence of DNA sequencing (shotgun, long-read, or single cell) and other omics technologies may play a crucial role in revealing strain-level variations and associated functional differences which are key to winegrowing and winemaking (32). Uncovering the fundamental biological mechanisms will additionally require truly integrative approaches, including high-throughput strain isolation and functional screening, assembly of synthetic communities, and their application in controlled fermentation experiments or gnotobiotic grapevine systems such as micropropagated plantlets (132, 152, 153). Results from such *in vitro* systems could be combined strategically with computational modeling to address remaining open questions such as the mechanistic basis of microbiome recruitment and community dynamics in grapevine.

Expanding interdisciplinary collaborations also represents a promising research direction. For example, most terroir and microbial terroir studies incorporate only a limited range of disciplines, e.g., focusing on geological, edaphic, plant phenology, or microbiological approaches within the scope of a single study (154); expanding to encompass all of these perspectives together will help mutually advance these fields and address broader questions such as the interaction of biological, microbiological, and abiotic factors on *terroir*. Moreover, the incorporation of anthropological perspectives could deepen our understanding of grapevine–microbiome systems by tracing how microbial communities have co-evolved with grapevine domestication and how they intersect with longstanding human cultural practices (155). To capture the complexity or viticultural systems in real-world settings will also require close collaborations with winegrowers and -makers. These stakeholders not only possess rich traditional knowledge but also should be actively involved in formulating relevant research questions and participative sampling strategies to reflect practical realities (52).

While bottlenecks and challenges remain, such as issues with low microbial biomass on grape berries which hinders shotgun sequencing, as well as the intrinsic complexity of genotype × environment × microbiome × management interactions remain, *microbial terroir* research offers valuable opportunities. Broader dissemination of this concept could provide stakeholders with a framework for identifying product-specific microbial signatures that support distinctiveness—an aspect still entirely absent from the economic literature on *terroir* (156). Microbiome research in viticulture and winemaking also has clear applied value for mitigating contamination risks, optimizing fermentation starters, and harnessing microbial diversity to enhance *terroir* expression (69, 157). In the vineyard, active microbiome management and application are still entirely nascent, yet hold immense potential for more sustainable viticultural practices (158, 159).

In summary, while *microbial terroir* should not be overemphasized as the sole driver of wine distinctiveness, it vastly broadens the current narrow focus on *Saccharomyces cerevisiae* in winemaking and pathogens in winegrowing. The composition and function of microbial communities are collectively shaped by the factors contributing to *terroir* and as such are a useful target to potentially improve wine production and advance sustainability in viticulture. Moreover, the dynamic nature of *microbial terroir* makes the grapevine microbiome a relevant model for studying ecological and evolutionary processes in a complex food ecosystem, with both practical and fundamental research applications.^159^
